# MicroRNA-133b Negatively Regulates Zebrafish Single Mauthner-Cell Axon Regeneration through Targeting *tppp3 in Vivo*

**DOI:** 10.3389/fnmol.2017.00375

**Published:** 2017-11-21

**Authors:** Rongchen Huang, Min Chen, Leiqing Yang, Mahendra Wagle, Su Guo, Bing Hu

**Affiliations:** ^1^Chinese Academy of Sciences Key Laboratory of Brain Function and Disease, School of Life Sciences, University of Science and Technology of China, Hefei, China; ^2^Programs in Human Genetics and Biological Sciences, Department of Bioengineering and Therapeutic Sciences, University of California, San Francisco, San Francisco, CA, United States

**Keywords:** axon regeneration, miR-133b, single-cell level, single-cell electroporation, *tppp3*, *in vivo* imaging

## Abstract

Axon regeneration, fundamental to nerve repair, and functional recovery, relies on rapid changes in gene expression attributable to microRNA (miRNA) regulation. MiR-133b has been proved to play an important role in different organ regeneration in zebrafish, but its role in regulating axon regeneration *in vivo* is still controversial. Here, combining single-cell electroporation with a vector-based miRNA-expression system, we have modulated the expression of miR-133b in Mauthner-cells (M-cells) at the single-cell level in zebrafish. Through *in vivo* imaging, we show that overexpression of miR-133b inhibits axon regeneration, whereas down-regulation of miR-133b, promotes axon outgrowth. We further show that miR-133b regulates axon regeneration by directly targeting a novel regeneration-associated gene, *tppp3*, which belongs to Tubulin polymerization-promoting protein family. Gain or loss-of-function of *tppp3* experiments indicated that *tppp3* was a novel gene that could promote axon regeneration. In addition, we observed a reduction of mitochondrial motility, which have been identified to have a positive correlation with axon regeneration, in miR-133b overexpressed M-cells. Taken together, our work provides a novel way to study the role of miRNAs in individual cell and establishes a critical cell autonomous role of miR-133b in zebrafish M-cell axon regeneration. We propose that up-regulation of the newly founded regeneration-associated gene *tppp3* may enhance axonal regeneration.

## Introduction

Axonal regeneration, critical for the maintenance of the nervous system, requires the coordinated expression of many regeneration-associated genes in the soma (Wu et al., 2012). Growing evidence indicates that microRNAs (miRNAs) play a crucial role during this process (Kloosterman and Plasterk, 2006; Strickland et al., 2011; Wu and Murashov, 2013; Li S. et al., 2016; Tedeschi and Bradke, 2017). MiRNAs are small, non-coding RNAs that function as negative regulators of gene expression, through imperfect base-pairing with the 3′-untranslated region (UTR) of target mRNAs thereby promoting mRNA degradation or inhibiting protein translation (Hong et al., 2014). Their ability to simultaneously regulate the expression of several genes suggests that miRNAs are crucial coordinators of complex gene expression programs.

Zebrafish exhibit high regenerative capacity in many tissues and organs, including heart muscles, spinal cord, sensory hair cells, appendages, and blood vessels (Stoick-Cooper et al., 2007). Moreover, many miRNAs have been implicated in these regenerative processes. For example, miR-101a regulates adult zebrafish heart regeneration (Beauchemin et al., 2015), and miR-10 regulates angiogenesis by affecting the behavior of endothelial cells (Hassel et al., 2012). MiR-133b, the miRNA of interest in this study, has been widely reported to participate in many regulatory processes. For example, miR-133b is considered as a tumor repressor in various human cancers, such as colorectal cancer (Hu et al., 2010; Akçakaya et al., 2011; Xiang and Li, 2014), gastric cancer (Wen et al., 2013), and gastrointestinal stromal tumor (Yamamoto et al., 2013). It also plays an important role in enhancing differentiation among different cell types, including muscle cells (Koutsoulidou et al., 2011) and neurons (Heyer et al., 2012). However, miR-133b exhibits different effects on different tissue regeneration. It has been shown to be a negative regulator in fin regeneration by targeting mps1 (Yin et al., 2008), while promoting spinal cord functional recovery after injury by targeting RhoA (Yu et al., 2011; Theis et al., 2017). Although, it also has been reported to promote neurite outgrowth at cellular level (Lu et al., 2015), its role, if any, in single-cell axon regeneration is not known.

*In vivo* imaging of single-axon regeneration in intact vertebrate is a powerful approach to gain mechanistic insights into this process (Kerschensteiner et al., 2005; Canty et al., 2013; Lorenzana et al., 2015; Xu et al., 2017). Although, previous studies have established miRNAs as crucial regulators in regenerative processes, little is known regarding their role in a single neuron during regeneration. Since nerve injury often associates with damages of both the nerve and neighboring tissues, it has been difficult to unveil autonomous vs. non-autonomous factors that influence axon regeneration *in vivo* (Rieger and Sagasti, 2011).

Using two-photo axotomy, a technology that can precisely injure a single axon (O'Brien et al., 2009; Canty et al., 2013; Xu et al., 2017), we have demonstrated that Mauthner-cells, a hindbrain neuronal type with large soma and long axons projecting toward the spinal cord, have the capacity to regenerate (Xu et al., 2017). In this study, we examined the role of miR-133b in M-cell regeneration. By single-cell electroporation and a vector-based expression system, we successfully altered the expression of miR-133b specifically in the M-cell. With a combination of gain-of-function and loss-of-function experiments, we demonstrated that miR-133b inhibits the regenerative process in M-cells. We further uncovered a novel regeneration-associated gene, *tppp3*, as a direct target of miR-133b in this process. Collectively, our findings identify a cell intrinsic mechanism involving miR-133b and its direct target *tppp3* in regulating axon regeneration *in vivo*.

## Materials and methods

### Animal care

Zebrafish (*Danio rerio*) WT/AB line was used in this study. Zebrafish embryos were maintained in embryo medium on a 14/10 light/dark cycle at 28.5°C. In case of the formation of pigment, 0.2 mM N-phenylthiourea (PTU, sigma) was added to the embryo medium at 24 h post fertilization (hpf). All animal manipulations were preformed strictly following the guidelines and regulations presented by the University of Science and Technology of China (USTC) Animal Resources Center and University Animal Care and Use Committee. The protocol was approved by the Committee on the Ethics of Animal Experiments of the USTC (Permit Number: USTCACUC1103013).

### Plasmids construction

To overexpress miRNAs, a construct containing pri-miR-133b/pri-miR-23a/pri-miR-21 was made by amplifying a genomic region containing the miR-133b/miR-23a/miR-21 precursor. The resulting PCR fragments were then inserted into the linearized pUAS-mCherry digested by NotI, locating at the 3′-UTR of mCherry.

To knock down miR-133b, we used the miRNA “sponge” assay, which presents an efficient and permanent miRNA loss-of-function by imperfectly binding to a miRNA of interest (Cohen, 2009). The plasmid pUAS-mcherry-8 × miR-133b sponge was designed by ourselves and then constructed by Sangon (Shanghai, China).

To generate overexpression of TPPP3 construct, full-length *tppp3* was initially amplified from complementary DNA (cDNA) of the WT/AB zebrafish strain. The PCR fragment was inserted into a plasmid backbone containing UAS. Plasmid UAS-tppp3 was co-delivered with both pUAS-mCherry and pCMV-Gal4-VP16 while electroporation.

ShRNA design was performed using the siRNA design tool under the following website: http://www.genscript.com/design_center.html (Dong et al., 2013). We selected the top five shRNAs (shRNA1-shRNA5) for further experiment. ShRNA expression vector was constructed in the following way: The modified mir30e backbone (Dong et al., 2013) was firstly synthesized with PacI-NheI sites for cloning target shRNA oligos. This modified mir30e precursor was cloned into pmini-Tol2-UAS-tdTOM vector downstream of tdTOM ORF to generate pmT2-UAS-tdTOM-mir30e-ShRNA (SG1180-A). We then cloned the fragment containing miR-shRNA structures (guide sequence, loop sequence, target sequence, and the flanking sequences) into pUAS-mCherry plasmid, locating in mCherry 3′-UTR. Target shRNA structures were synthesized by Sangon (Shanghai, China) and then cloned into PacI-NheI site.

### Microinjection and quantitative real-time PCR

One-cell stage zebrafish embryos were injected with a solution consisting of 30 ng/μl CMV-Gal4-VP16 plasmid and 30 ng/μl pUAS-mCherry/pUAS-mCherry-mircoRNA/pUAS-mCherry-miR-shRNA. To detect miRNAs level, 3 days post fertilization (dpf) zebrafish larvae with relatively high mosaic red fluorescence were selected for total RNAs isolation by miRNA Isolation Kit (Tiangen), according to the manufacturer's protocols. Each sample was reverse-transcribed into cDNA by miRNA First-Strand cDNA Synthesis Kit (Tiangen) and was subjected to qRT-PCR analysis with qPCR Detection Kit (Tiangen). To detect mRNAs levels, 10 hpf zebrafish embryos expressing red fluorescence were selected to isolate total RNAs with the same kit mentioned above. Each sample was reverse-transcribed into cDNA with HiScriptII Q RT SuperMix (Vazyme) and was subjected to qRT-PCR analysis with AceQ qPCR SYBR Master Mix (Vazyme). Each experiment was carried out with three biological and experimental replicate. Results were shown as mean fold changes ±s.e.m. qRT–PCR primers were shown in Table S1.

### Single-cell electroporation

Before electroporation, 4 dpf zebrafish larvae were embedded in 1% low-melting agarose gel on an electroporation chamber. Using a micropipette (WPI, USA) pulled by a micropipette puller (P-97, Sutter, USA) to electroporate plasmids into the M-cell soma by pushing the tip against it with a series of pulses at 14–16 V. CMV-Gal4-VP16 plasmid was co-delivered into the unilateral M-cell of zebrafish larva with pUAS-mCherry-microRNAs (plasmids used to overexpress specific miRNA)/pUAS-mCherry-microRNA sponge (plasmid used to inhibit specific miRNA)/pUAS-mCherry-miR-shRNA (plasmid used to inhibit *tppp3*). Each plasmid concentration is 120 ng/μl. Zebrafish electroporated with pCMV-Gal4-VP16 and pUAS-mCherry were treated as control. For the experiment to overexpress TPPP3, pUAS-tppp3 was delivered into cell soma with both pCMV-Gal4-VP16 and pUAS-mCherry. After electroporation, larvae were returned back to embryo medium containing PTU. Then we selected morphologically healthy zebrafish expressing red fluorescence in M-cells for later experiment.

### Two-photon axotomy

Before axotomy, 6 dpf zebrafish larvae expressing red fluorescence in unilateral M-cells were anesthetized in MS222 (Sigma, USA) and fixed in 1% low-melting agarose. A Zeiss microscope (LSM710, Germany) was used to ablate the M-cell axons over cloacal pores. We normally set the 800 nm two-photon laser at an intensity of 12–15% to damage axon over ~1.5 s (Xu et al., 2017).

### *In Vivo* imaging and data analysis

Before imaging, embryos were anesthetized by MS222 and then embedded in 1% low melting point agarose in embryo medium containing MS222. All images and time-lapse movies were taken from lateral views of the spinal cord, anterior to the left, and dorsal toward the top.

To observe M-cells regrowth after ablation at 6 dpf, anesthetized zebrafish were imaged at 1–2 days post-axotomy (dpa) using Olympus FV1000 confocal microscope (Olympus, Tokyo, Japan) equipped with a 40x, 0.8 N.A. water-immersion objective at 2-μm intervals. All images well spliced using with Photoshop CS4 (Adobe, USA). We defined the starting point of regrowth as the ablated site of axons just above cloacal pores, and the axonal terminal of regeneration was stipulated as the end point of regrowth axons. In this article, regeneration length refers to the maximum regenerated axon length of one branch, while total regeneration length refers to all the regenerated axon branches length combined. All regenerative length was calibrated to convert pixels into distance using FV10-ASW 4.2 viewer software.

For investigating mitochondrial transport in single M-cell *in vivo*, zebrafish larva electroplated with pUAS-mito-eGFP (plasmid used to label mitochondria) were imaged at 6 dpf using a confocal microscope with a 60x, 0.9 N.A. water-immersion objective. 2.5-min movies of the axonal area, locating within 200 nm proximal to the site above the cloacal pores, were taken with an imaging frequency about 1.5 s, and the imaging length of axons was ~43 mm at the site of the axon. All images were processed with Fiji/ImageJ (National Institutes of Health, USA). The quantification of mitochondrial dynamics were measured as previously described (Misgeld et al., 2007; Plucinska et al., 2012; Takihara et al., 2015; Xu et al., 2017). Mitochondrial motility was defined as the percentage of moving mitochondria, which were identified to move more than 2 μm, during the 2.5-min time-lase movies. The velocity of a moving mitochondrion referred to the total moving distance of a mitochondrion divided by its observed moving time.

### EGFP sensor assay

*In vitro* transcription of EGFP-tppp3 3′-UTR, EGFP-tppp3 mut-3′-UTR and mCherry mRNAs were performed with mMESSAGE mMACHINE T7 Ultra Kit (Invitrogen) and these synthesized mRNAs were purified with MEGAclearTM Kit (Invitrogen). Zebrafish embryos at one-cell stage were injected with a combing solution of sensor mRNA and mCherry mRNA. When applicable, 10 μM miR-133b duplex was added as an experimental group, while 10 μM non-sense duplex was added as a control. EGFP fluorescence was quantified at 24–28 h post-fertilization (hpf) using software Fiji-imageJ.

### Statistical analysis

The distribution of data points was expressed as mean ± standard error of the mean (S.E.M.), or as relative proportion of 100% as mentioned in the appropriate legends. Depending on the number of the groups and independent factors, student's *t*-tests, one-way analyses of variance (ANOVA) and non-parametric tests were used as indicated in the figures. Results were classed as significant as follows: ^*^P < 0.05, ^**^P < 0.01, and ^***^P < 0.001.

## Results

### Overexpression of miR-133b in single M-cell inhibits axon regeneration

**Figure 1:**
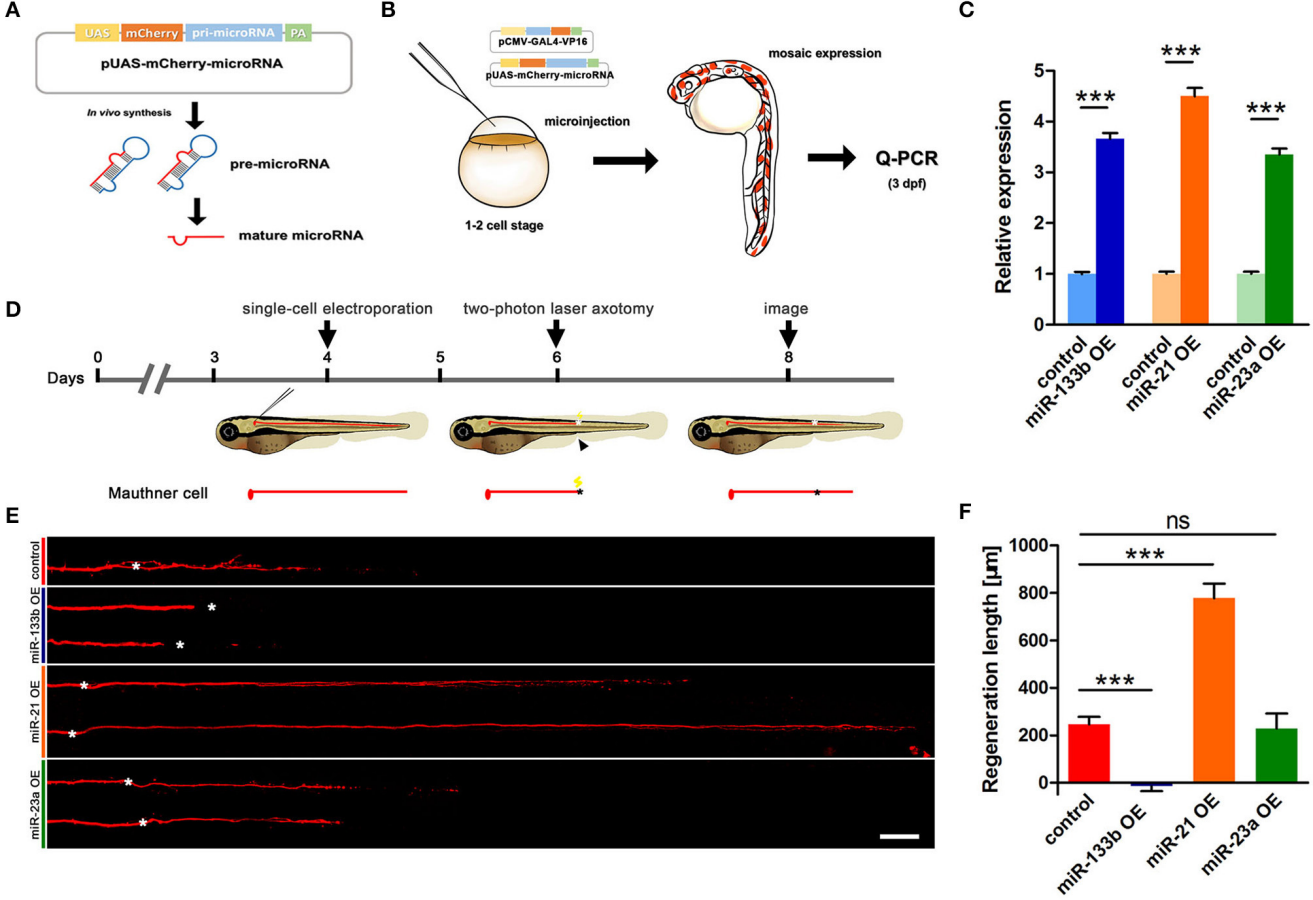
Vector-based overexpression of miR-133b by single-cell electroporation inhibits M-cell regeneration. **(A)** Construction of the vector-based microRNA expression system. Plasmids express only mCherry served as control vector. **(B)** Validation of the vector-based expression system. Choose the larvae at 3 dpf with relatively high mosaic expression to detect the expression of microRNA. **(C)** Quantitative RT-PCR analysis exhibited an increase of different miRNA levels by vector-based expression *in vivo*. The control vector-injected embryos served as controls. **(D)** Design for microRNA-expression vector electroporation studies. Axons of Mauthner-cell labored with red fluorescent was ablated at 6 dpf right above the cloacal pores (black arrow), and confocal image at 8 dpf (2 dpa). Black asterisk: ablation point. **(E)** Confocal imaging of M-cells expressing different miRNAs at 2 dpa. White asterisk: ablation point. Scale bar: 50 μm. **(F)** Regeneration length at 2 dpa. One-way ANOVA, *P* < 0.0001: Student's two-tailed *t*-test, control vs. miR-133b OE, *P* < 0.0001; control vs. miR-21 OE, *P* < 0.0001; control vs. miR-23a OE, *P* = 0.8312. ^*^P < 0.05, ^***^P < 0.001. Error bars represent S.E.M.

We have identified in our previous study that M-cells have strong regenerative capacity (Xu et al., 2017). More than 90% of two-photo ablated M-cells could regenerate a certain length in our experiments. To explore the role of miR-133b in M-cell axon regeneration, we performed cell type-specific overexpression. A vector-based miRNA expression was used to achieve enduring expression of the miRNA during our experimental time window. We constructed a vector containing dre-pri-miR-133b sequence (miRBase Accession: MI0001994) in the 3′-UTR of mCherry, which conveniently marked the cells that expressed the miR-133b (Figure 1A). The plasmid UAS-mCherry-miR-133b was co-injected with pCMV-Gal4-VP16 into one-cell zebrafish embryos. As a control, embryos were injected with pUAS-mCherry and pCMV-Gal4-VP16. We then selected zebrafish larvae with relatively high mosaic red fluorescence at 3 dpf to isolate the total RNA (Figure 1B). Our qRT-PCR data showed that miR-133b in experimental group (EG) was more than three times of that in control, indicating that our constructed plasmid UAS-mCherry-miR-133b could successfully drive overexpression of miR-133b (Figure 1C).

Next, we used this vector system to overexpress miR-133b in individual M-cells at 4 dpf via single-cell electroporation. We selected the zebrafish with red fluorescence in unilateral M-cell at 6 dpf for two-photon laser axotomy and visualized axon regeneration at 2 dpa (Figure 1D). Our imaging data showed that most M-cells in control could regenerate a certain length, while M-cell overexpressing miR-133b could hardly regenerate [control: 243.7 ± 32.9 μm, *n* = 33 fish vs. miR-133b overexpression (OE): −14.8 ± 20.7 μm, *n* = 16 fish] (Figures 1E,F). To further verify the specific role of miR-133b in regulating axon regeneration, we overexpressed another two miRNAs, miR-23a and miR-21, with the same assay as mentioned above. Together with qRT-PCR results confirming that miR-23a and miR-21 were indeed overexpressed in zebrafish via vector-based miRNA expression assay (Figure 1C), we found out that miR-23a, a miRNA that has not been reported to be associated with axon regeneration, had no obvious effect on M-cell axon regeneration; while miR-21, which has been shown to promote regeneration in different organs (Strickland et al., 2011; Han et al., 2014; Hoppe et al., 2015), remarkably promoted M-cell axon regeneration (control: 243.7 ± 32.9 μm, *n* = 33 fish vs. miR-23a OE: 229.0 ± 62.4 μm, *n* = 10 fish vs. miR-21 OE: 778.4 ± 60.8 μm, *n* = 12 fish; Figures 1E,F).

Since researches on dre-miRNAs often explore their roles in different processes using miRNA duplex, to further verify miR-133b's role on axon regeneration, we also expressed the miR-133b duplex in M-cell by single-cell electroporation. M-cells expressing only rhodamine-dextran (3,000 molecular weight, Invitrogen) (named None) seemed to have similar outgrowths to those expressing non-sense duplex (named Negative Control), while both explicated a slight increase, even though without significant discrepancy, compared to M-cells in experimental group (expressing miR-133b duplex), not matter at 1 dpa or 2 dpa (1 dpa: None: 142.3 ± 19.0 μm, *n* = 26 fish; Negative control: 140.0 ± 15.8 μm, *n* = 24 fish; miR-133b duplex: 102.7 ± 17.6 μm, *n* = 23 fish; 2 dpa: None: 464.8 ± 40.5 μm, *n* = 20 fish; Negative control: 396.7 ± 32.5 μm, *n* = 22 fish; miR-133b duplex: 373.4 ± 33.9 μm, *n* = 23 fish; Figure S1). This result was consistent with the results obtained by vector-based system, indicating that miR-133b has negatively effects on M-cell axon regeneration. Together, these results indicate that the reduction of M-cell regenerative capability by miR-133b is specific and cell intrinsic.

### Impairment of miR-133b function in M-cell promotes axon outgrowth

To determine whether loss of miR-133b in single M-cells could also regulate its axon regeneration, we needed an assay that could achieve long-term miRNA loss-of-function. MiRNA sponges have been shown to efficiently bind to endogenous miRNAs and block their silencing activity with bulged miRNA binding sites (Ebert et al., 2007; Cohen, 2009; Otaegi et al., 2011). Moreover, the bulged sites can protect against cleavage and degradation of sponge RNA by the Ago2 component of the RISC (Ebert et al., 2007; Ebert and Sharp, 2010), which can satisfy our experimental requirement.

**Figure 2:**
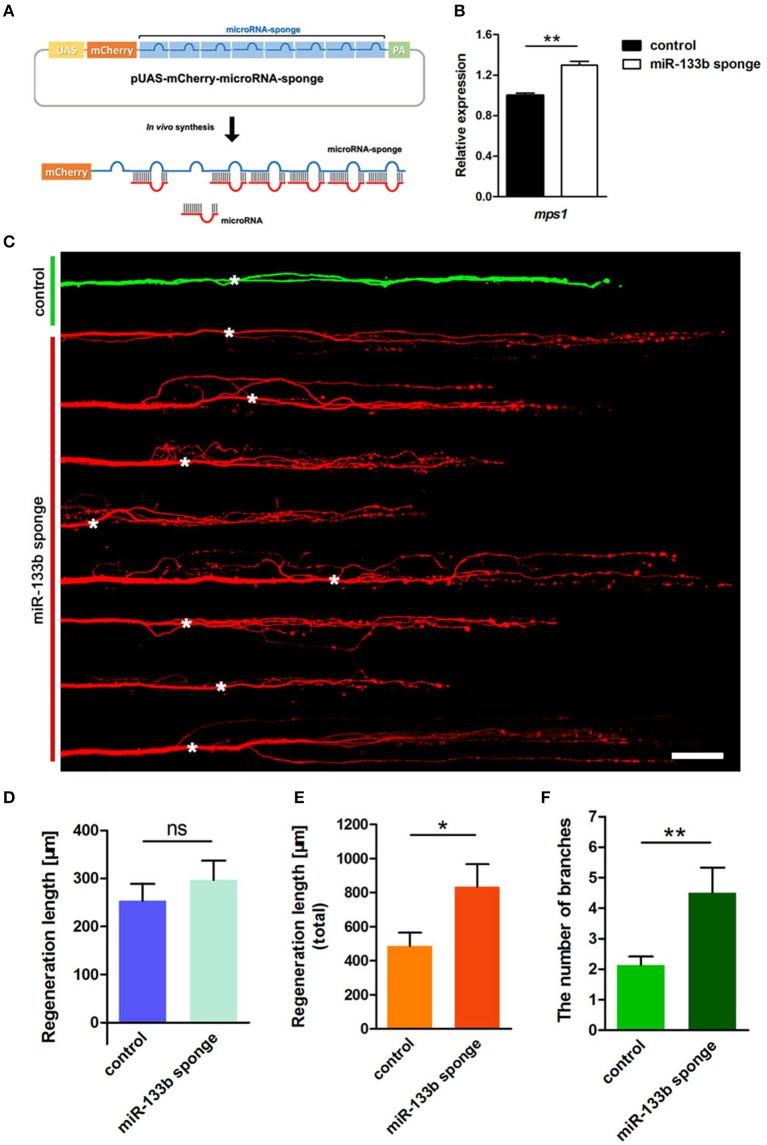
Knockdown of miR-133b by expressing miR-133b sponge facilitates M-cell regeneration. **(A)** Design of miRNA sponges. The construction of miRNA sponges was manipulated by inserting multiple microRNA binding sites in the 3′-UTR of the mcherry reporter gene. Plasmids express only mCherry served as control vector. **(B)** Quantitative RT-PCR analysis exhibited an increase in *mps1* mRNA expression in 10 hpf zebrafish embryos by miR-133b sponge expression *in vivo*. **(C)** Confocal imaging of M-cell at 2 dpa. White asterisk: ablation point. Scale bar: 50μm. **(D)** Regeneration length at 2 dpa. Student's two-tailed *t*-test, *P* = 0.4300. **(E)** Total regeneration length at 2 dpa. Student's two-tailed *t*-test, *P* = 0.0194. **(F)** The number of branches at 2 dpa. Non-parametric tests, *P* = 0.0047. ^*^P < 0.05, ^**^P < 0.001. Error bars represent S.E.M.

We constructed a plasmid containing 8 bulged target sites complementary to miR-133b in 3′-UTR of mCherry reporter gene driven by the UAS promoter (Figure 2A). To testify the ability of this plasmid in blocking miR-133b activity in zebrafish, we examined the expression of a known miR-133b target gene, *mps1* (Yin et al., 2008), in 10 hpf zebrafish embryos injected with a combination of pUAS-mCherry-8 × miR-133b sponge and pCMV-GAL4-VP16 at one-cell stage. The *mps1* mRNA level increased in zebrafish larvae expressing the miR-133b sponge, suggesting that it could reduce miR-133b activity in zebrafish (Figure 2B).

Next, we examined the consequence of knocking down miR-133b activity in axon regeneration. Remarkably, most axons regenerated with supernumerary branches (Figure 2C). The longest regeneration length of a single axon had no significant difference between control and experimental group (control: 253.7 ± 34.9 μm, *n* = 30 fish vs. miR-133b sponges: 296.7 ± 40.5 μm, *n* = 20 fish; Figure 2D). However, the total regeneration length, all branches combined, was significantly different (control: 486.7 ± 78.8 μm, *n* = 30 fish vs. miR-133b sponges: 835.2 ± 131.4 μm, *n* = 20 fish; Figure 2E). The experimental group had significantly more axonal branches than the control (control: 2.13 ± 0.28, *n* = 30 fish vs. miR-133b sponges: 4.50 ± 0.83 μm, *n* = 20 fish; Figure 2F). Collectively, these results demonstrate that blocking the function of miR-133b promotes M-cell axon outgrowth, which is a phenotype that is complementary to overexpressing miR-133b in M-cells.

### *Tppp3* is an *in Vivo* target of miR-133b

**Figure 3:**
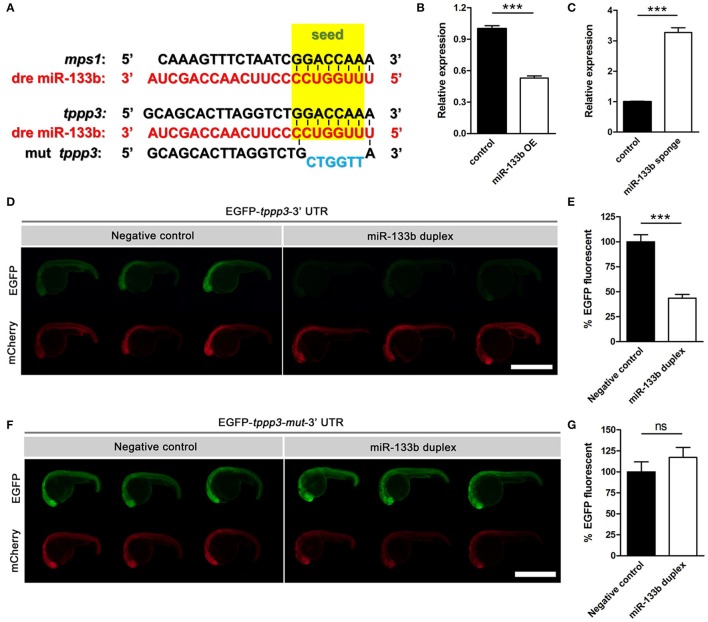
Sequence alignment and the EGFP sensor assay show that miR-133b targets *tppp3*. **(A)** Sequence alignment of zebrafish miR-133b, *tppp3* 3′UTR and its mutation version (within the 2–7 nt mutated) was shown, with the seed sequences highlighted in yellow box and the mutational nucleotides in blue. *mps1* 3′UTR was shown as a control. **(B)** Quantitative RT-PCR analysis exhibited a decrease in *tppp3* mRNA expression in 10 hpf zebrafish embryos by the vector-based miR-133b overexpression *in vivo*. **(C)** Quantitative RT-PCR analysis exhibited an increase in *tppp3* mRNA expression in 10 hpf zebrafish embryos by miR-133b sponge expression *in vivo*. **(D)** EGFP-*tppp3* 3′UTR shown strong fluorescent signals when co-injected with non-sense duplex (as negative control), but failed to give fluorescent signals when co-injected with miR-133b duplex. mCherry mRNA was injected as a control. **(E)** The EGFP-*tppp3* 3′UTR fluorescence was expressed as a percentage of fluorescent signal observed from the negative control. Student's two-tailed *t*-test, *P* < 0.0001. *n* = 10 for each group. **(F)** Both groups shown strong fluorescent signals whenever the EGFP-*tppp3* mut-3′UTR coinjected with the miR-133b duplex or non-sense duplex. **(G)** The EGFP-*tppp3* mut-3′UTR fluorescence was expressed as a percentage of fluorescent signal observed from the negative control. Student's two-tailed *t*-test, *P* = 0.3219. *n* = 10 for each group. ^***^P < 0.001. Error bars represent S.E.M.

Typically, one miRNA can suppress the expression of many genes by interacting with the 3′-UTR or the coding regions of the targets mRNAs (Lewis et al., 2005; Duursma et al., 2008; Forman et al., 2008). We searched several databases, including TargetScan Fish, miRBase and microcosm Targets, and identified potential targets containing complementary regions to miR-133b seed sequences in their 3′-UTR. We focused on one gene, *tppp3*, which has a single binding site for miR-133b at its 3′-UTR. In addition, *tppp3* corresponds perfectly to nucleotides 2–7 of the mature miR-133b in zebrafish (Figure 3A). TPPP3 is a member of tubulin polymerization promoting protein family. Previous studies identified TPPP3 as a potent inducer of tubulin polymerization (Vincze et al., 2006) and human TPPP3 binds and stabilizes microtubules (MTs; Oláh et al., 2017). Since regulation of axonal microtubule (MT) dynamics influence axon regeneration (Sengottuvel and Fischer, 2011; Bradke et al., 2012; Hur et al., 2012), and pharmacological stabilization of MTs by paclitaxel or related molecules promotes axon regeneration *in vitro* and *in vivo* (Hellal et al., 2011; Sengottuvel et al., 2011; Ruschel et al., 2015), we hypothesized that miR-133b might regulate axon regeneration through directly modulating *tppp3* mRNA *in vivo*.

We firstly detected the mRNA level of *tppp3* in miR-133b overexpressed or miR-133b sponge expression zebrafish embryos. Our qRT-PCR results showed that the mRNA level of *tppp3* in 10 hpf zebrafish embryos overexpressing miR-133b was lower than that in control (Figure 3B), while *tppp3* mRNA level was increased in embryos expressing miR-133b sponge compared with that in control (Figure 3C). We then used zebrafish embryo sensor assays (Giraldez et al., 2005). Two mRNAs were synthesized, one encoding enhanced green fluorescent protein (EGFP) with 3′- UTR of *tppp3* and the other composed of mCherry fluorescent protein with a poly(A) alone. These mRNAs were co-injected into one-cell zebrafish embryos, in the presence of miR-133b RNA duplex or non-sense duplex (GenePharma). Injections of these two mRNAs along with a non-sense RNA duplex (negative control) resulted in both high EGFP expression and mCherry expression. However, when a synthesized duplex of miR-133b was co-injected, EGFP signals were dampened by almost 50% with no detective changes in mCherry signals (negative control: 100.0 ± 7.0%, *n* = 10 fish vs. miR-133b duplex: 43.5 ± 3.7%, *n* = 10 fish; Figures 3D,E). When the seed sequence in the 3′-UTR of *tppp3* was mutated, we found no difference in EFGP signals between non-sense RNA duplex and miR-133b RNA duplex (negative control: 100.0 ± 11.9%, *n* = 10 fish vs. miR-133b duplex: 117.1 ± 12.0%, *n* = 10 fish; Figures 3F,G).

In conclusion, our results indicate that *tppp3* is a downstream gene of miR-133b *in vivo*.

### TPPP3 is critical to enhance axonal outgrowth

**Figure 4:**
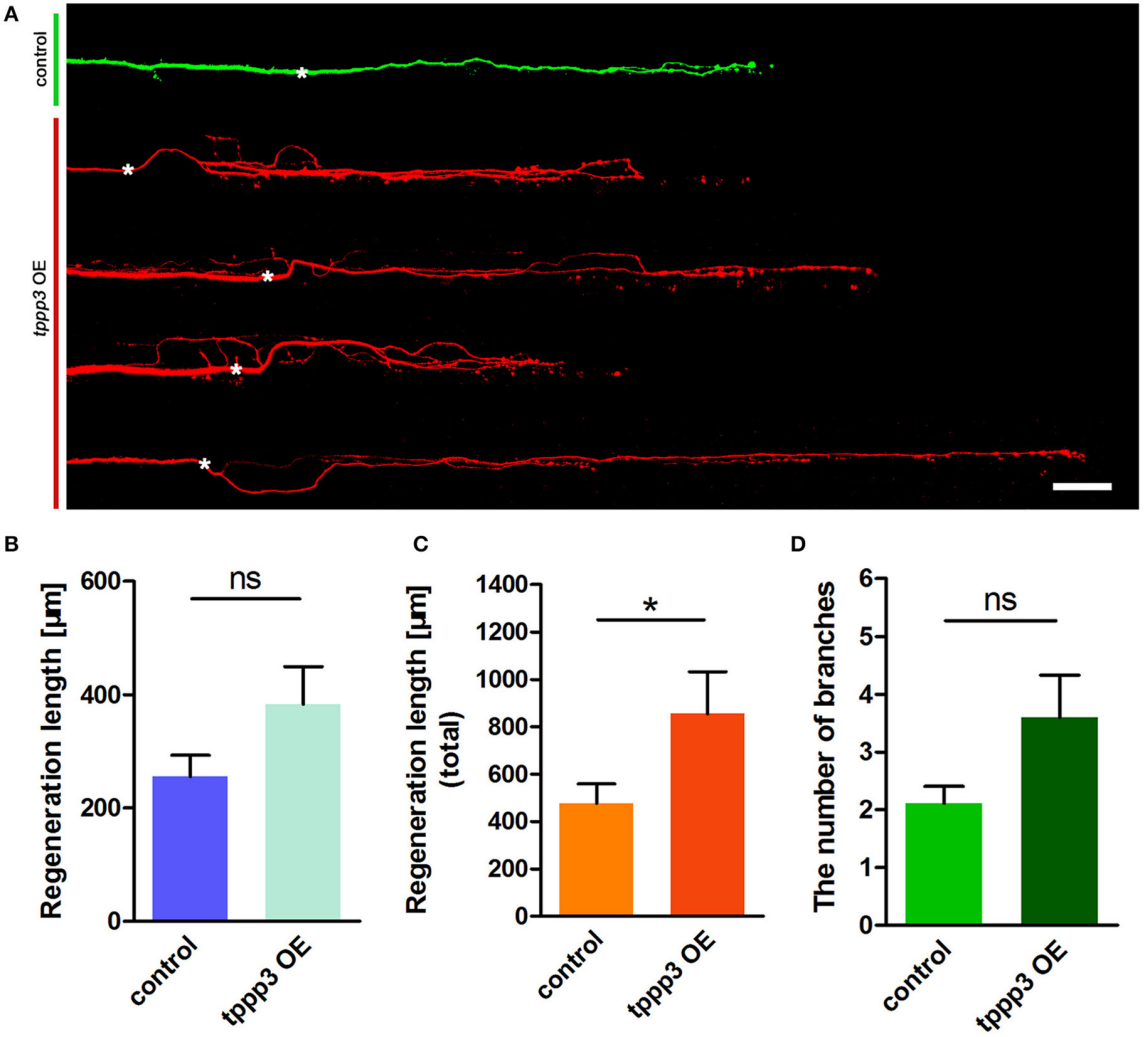
Overexpression of *tppp3* by single-cell electroporation promotes M-cell regeneration. **(A)** Confocal imaging of M-cell at 2 dpa. White asterisk: ablation point. Scale bar: 50 μm. **(B)** Regeneration length at 2 dpa. Student's two-tailed *t*-test, *P* = 0.0775. **(C)** Total regeneration length at 2 dpa. Student's two-tailed *t*-test, *P* = 0.0338. **(D)** The number of branches at 2 dpa. Non-parametric tests, *P* = 0.0516. ^*^P < 0.05. Error bars represent S.E.M.

Given the effects of miR-133b on *tppp3* expression and the role of miR-133b in neurite outgrowth, we next planned to investigate the effects of gain or loss-of-function of *tppp3* on regenerative axon growth. We firstly overexpressed *tppp3* by electroplating into M-cell at 4 dpf a plasmid containing the zebrafish *tppp3* cDNA. As a control, pUAS-mcherry and pCMV-GAL4 was delivered. Consistent with the effects of miR-133b sponge on axonal regeneration (Figure 4A), overexpression of *tppp3* in M-cell significantly increased the total regeneration length (Regenerative length: control: 255.6 ± 37.2 μm, *n* = 27 fish vs. TPPP3 OE: 382.9 ± 66.6 μm, *n* = 15 fish; total regeneration length: control: 476.2 ± 83.2 μm, *n* = 27 fish vs. TPPP3 OE: 855.2 ± 177.4 μm, *n* = 15 fish; Figures 4B,C), although there is no significant difference in branching number (control: 2.11 ± 0.29, *n* = 27 fish vs. TPPP3 OE: 3.60 ± 0.73, *n* = 15 fish; Figure 4D).

**Figure 5:**
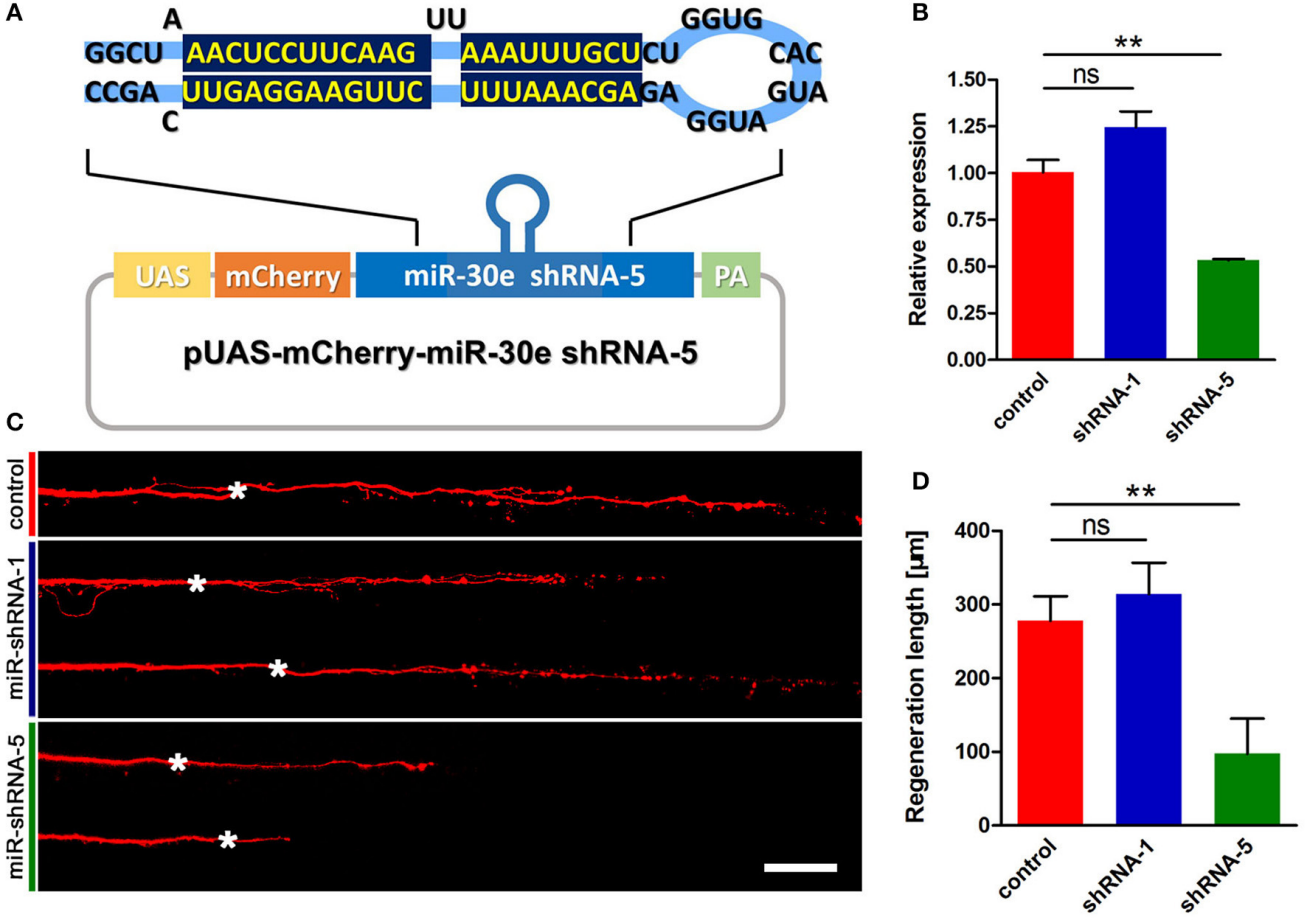
miR-shRNA based gene silence of *tppp3* diminishes regenerative length of M-cell **(A)** Diagram of miR-shRNA system. Sequence of ShRNA-5 was presented here, with the guide strand (bottom) highlighted in dark blue. **(B)** Quantitative RT-PCR analysis exhibited a deduction of *tppp3* mRNA in shRNA-5 expressing embryos. **(C)** Confocal imaging of M-cell at 2 dpa. White asterisk: ablation point. Scale bar: 50 μm. **(D)** Regeneration length at 2 dpa. Student's two-tailed *t*-test, control vs. shRNA-1, *P* = 0.5807; control vs. shRNA-5, *P* = 0.0025. ^**^P < 0.01. Error bars represent S.E.M.

To test whether knockdown of *tppp3* might cause regenerative defects similar to miR-133b overexpression, we used designed shRNAs to silence *tppp3* based on the miR-ShRNAs system. MiR-shRNAs have now been widely used in mammals and zebrafish (De Rienzo et al., 2012; Dong et al., 2013; Shinya et al., 2013), *in vitro* and *in vivo* (Giraldez et al., 2005; Zuber et al., 2011), due to its higher efficiency than simple hairpin designs. We designed shRNAs employing the primary miR-30 backbone. Based on the Web-based shRNA design tool (https://www.genscript.com), five shRNAs (shRNA1-shRNA5) targeting the *tppp3* gene were selected (Figure S2). mCherry was used as a fluorescent reporter to mark the zebrafish embryos that expressed the miR-shRNA (Figure 5A). To valid the function of these shRNAs, we injected the miR-shRNA expressing plasmids combing with pCMV-GAL4 into one-cell stage embryos and isolated mRNA of these embryos exhibiting red fluorescence at 10 hpf to examine the *tppp3* mRNA level. We found that, among these five shRNAs, shRNA-5 exhibited the significant reduction of *tppp3* mRNA level (Figure 5B). We then investigated the effects of shRNA-5 on axonal regeneration by delivering it into M-cells via single-cell electroporation at 4 dpf. To avoid the effects of other miR-shRNA structures (guide sequence, loop sequence, and the flanking sequences) on the capability of regeneration, cells expressing shRNA-1, which had little effects on reducing *tppp3* mRNA (Figure 5B), were used as an additional control. Both imaging and quantitative results indicated that shRNA-5 diminished the regenerative length of damaged axons, while shRNA-1did not (control: 278.2 ± 33.1 μm, *n* = 26 fish vs. miR-shRNA-1: 314.2 ± 42.7 μm, *n* = 8 fish vs. miR-shRNA-5: 97.6 ± 47.7 μm, *n* = 20 fish; Figures 5C,D).

Taken together, these results indicate that *tppp3* is critical to promote axon outgrowth.

### Mir-133b attenuates mitochondrial motility in M-cell

Mitochondria plays a critical role in axon regeneration, a highly energy-demanding process. Our previous study has indicated that mitochondrial trafficking is associated with axon regenerative capacity, suggesting that axons having more motile mitochondria regenerate better than those having less ones (Xu et al., 2017). Moreover, another research group finds out that mature injured axons in mice can regenerate by enhancing mitochondrial motility via genetic manipulation, which helps remove damage mitochondria and recruit new ones to meet the energy demands at injury sites during regenerative process (Zhou et al., 2016).

**Figure 6:**
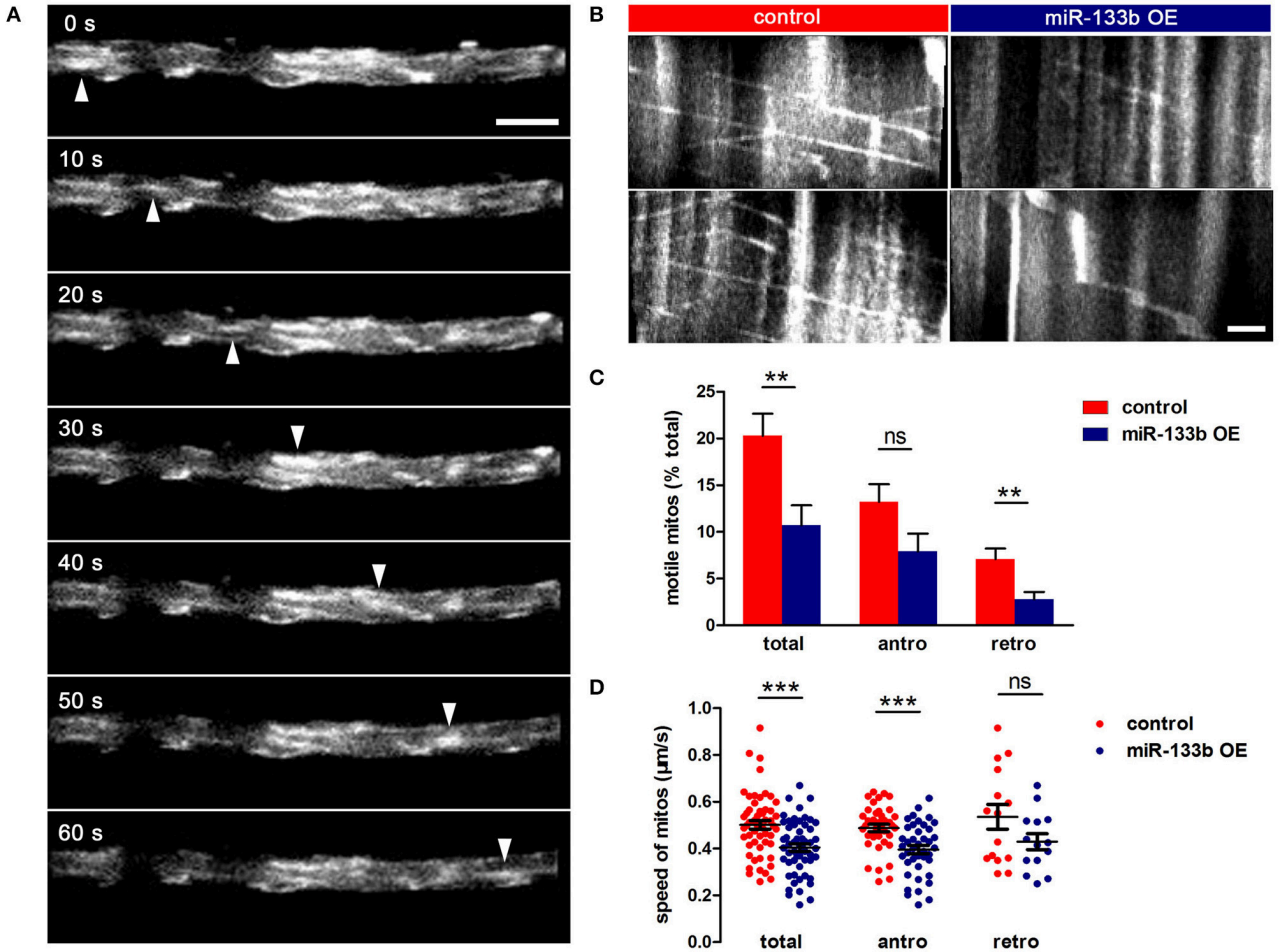
miR-133b attenuates mitochondrial transport in M-cell. **(A)** *In vivo* time-lapse sequences showing a moving mitochondrion (white arrowhead). Scale bar: 5 μm. **(B)** Kymographs depict mitochondrial movement in control (left) and miR-133b OE group (right) at 6 dpf. Scale bar: 5μm. **(C)** Comparison of mitochondrial motility in control and miR-133b OE group, including total, anterograde and retrograde. Student's two-tailed *t*-test, total: control vs. miR-133b OE, *P* = 0.0063; antero: control vs. miR-133b OE, *P* = 0.0640; retro: control vs. miR-133b OE, *P* = 0.0038. **(D)** Comparison of mitochondrial moving speed in control and miR-133b OE group, including total, anterograde, and retrograde. Student's two-tailed *t*-test, total: control vs. miR-133b OE, *P* = 0.0001; antero: control vs. miR-133b OE, *P* = 0002; retro: control vs. miR-133b OE, *P* = 0.1081, ^**^P < 0.01, ^***^P < 0.001. Error bars represent S.E.M.

To examine whether miR-133b overexpression had any effects on mitochondrial dynamics, we co-transfected pUAS-mito-EGFP and pUAS-mcherry-miR-133b driven by the expression of pCMV-GAL4 via single-cell electroporation at 4 dpf and visualized the movement of mitochondria at 6 dpf via *in vivo* time-lapse confocal imaging, through which stable vs. mobile mitochondria could be discerned (Figure 6A, Video S1). By counting and analyzing mitochondria in M-cells, we identified that the percentage of motile mitochondria was much lower in miR-133b overexpressing conditions than in control (Figure 6B, Video S2), and this reduction was more significant in retrograde than in anterograde directions (Total: control: 20.31 ± 2.34%, *n* = 11 fishes vs. miR-133b OE: 10.70 ± 2.14%, *n* = 13 fishes; antero: control: 13.21 ± 1.89%, *n* = 11 fishes vs. miR-133b OE: 7.91 ± 1.92%, *n* = 13 fishes; retro: control: 7.10 ± 1.11%, *n* = 11 fishes vs. miR-133b OE: 2.79 ± 0.77%, *n* = 13 fishes; Figure 6C). Moreover, mitochondrial velocity in the miR-133b overexpression group was slower in both transport directions compared with that in control, though in retrogradely moving mitochondria it did not reach significance (Total: control: 0.501 ± 0.018 μm/s, *n* = 54 mitos from 11 fishes vs. miR-133b OE: 0.404 ± 0.015 μm/s, *n* = 55 mitos from 13 fishes; antero: control: 0.488 ± 0.015 μm/s, *n* = 39 mitos from 11 fishes vs. miR-133b OE: 0.396 ± 0.017 μm/s, *n* = 41 mitos from 13 fishes; retro: control: 0.535 ± 0.052 μm/s, *n* = 15 mitos from 11 fishes vs. miR-133b OE: 0.4294± 0.034 μm/s, *n* = 14 mitos from 13 fishes; Figure 6D). Together, our results suggest that miR-133b is an important cell intrinsic regulator of mitochondrial dynamics during M-cell axon regeneration.

## Discussion

Through modulating miRNA in single neuron and *in vivo* imaging, we have made several new findings in this study. First, using Mauthner cells as the model, we demonstrate, through both loss and gain-of-function experiments, a critical cell-intrinsic role of miR-133b in inhibiting axon regeneration. Second, we uncover a previously unknown molecular target of miR-133b, *tppp3*, and show that it is a critical cell-intrinsic factor in promoting axon outgrowth. Finally, we reveal that miR-133b negatively regulates mitochondrial dynamics, which further supports the negative effects of miR-133b on axon regeneration.

**Figure 7:**
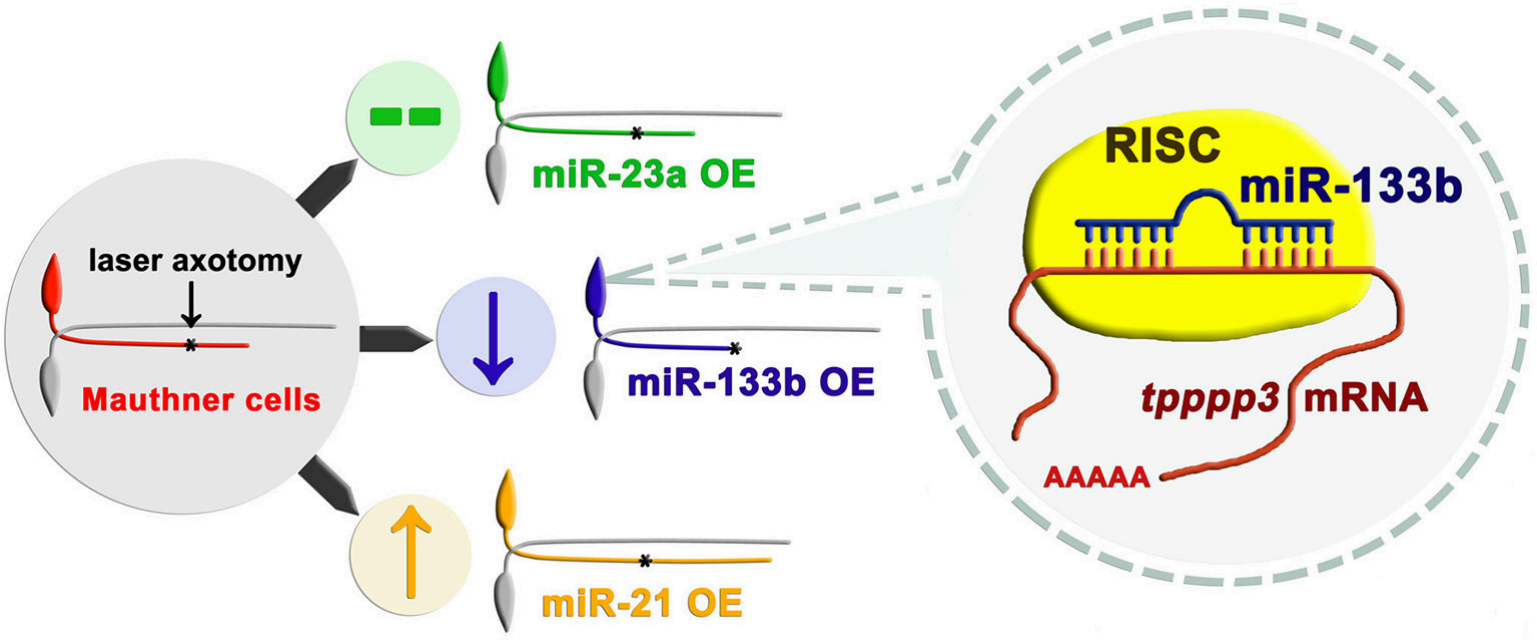
Working model of how miR-133b involved in regulating axonal regeneration of M-cell. Different miRNAs play different roles on axonal regeneration. MiR-133b modulates M-cell regenerative capacity via diminishing *tppp3* mRNA level, a novel gene that regulates axon outgrowth. Black asterisk: ablation point.

Maunther cells, a pair of myelinated neurons with large soma and a long axon extending from hindbrain to tail in zebrafish, have been proved to have regenerative capacity in our previous study (Xu et al., 2017). Distinct from conventional miRNA over-expression system in zebrafish with the RNA duplex, our study used a vector-based system that enabled long-term expression of miRNAs. With another two miRNAs (miR-23a and miR-21) having different effects on axon regeneration, we reported that overexpression of miR-133b specifically reduced the regenerative length in M-cell (Figure 7). To further verify the validity of our vector-based system, we also delivered the miR-133b duplex into M-cell via single-cell electroporation. MiR-133b duplex delivered group exhibits a reduction tendency in axon regeneration length, although without a significant change, which might be due to the application of low dose of RNA duplex during single-cell electroporation compared with that in microinjection. What's more, we found that this tendency seems shrunk at 2 dpa, which might be due to a degradation of miR-133b duplex. Combining with the results of axon outgrowth in miR-133b sponge group, we identified the negative role of miR-133b during M-cell regenerative process.

We have further identified *tppp3* as the target of miR-133b in regulating axonal regeneration in zebrafish M-cell (Figure 7). The direct interaction between miR-133b and *tppp3* mRNA was confirmed by EGFP sensor assay. *Tppp3* expression was down-regulated by miR-133b at the mRNA level. Although we did not detect the change at protein level of *tppp3* because of the limitation of antibody performing in zebrafish, it did not cast much doubts on the credibility that *tppp3* is a downstream gene of miR-133b *in vivo*. At the same time, our data do not exclude possibility that there is another gene that is regulated by miR-133b in this process too.

*Tppp3* was originally discovered as a member of the tubulin polymerization-promoting family that induces tubulin polymerization and has been extensively studied recently (Vincze et al., 2006; Staverosky et al., 2009; Juneja, 2013; Orosz, 2015). Researches has identified its critical role on promoting proliferation and preventing apoptosis *in vitro* (Zhou et al., 2010; Li Y. et al., 2016). Moreover, there is a study confirms its expression in motor neuron and suggests it may play a role in regulating sensory neuron regeneration in zebrafish (Aoki et al., 2014). Although, there has been no direct evidence demonstrating that *tppp3* can promote regeneration, a mount of studies confirms that microtubule stability, which has been identified to be one role of *tppp3* in human, is crucial to improve regenerative capability. Thus, concerning with the highly evolutional conservation of *tppp3* between human and zebrafish (Orosz, 2012; Oláh et al., 2017), which indicates that there may be a functional similarity between them, we speculate *tppp3* may involve in promoting axon regeneration in zebrafish M-cells. In our study, *tppp3* gain or loss-of-function produced a regulation on axon outgrowth mimicking the effect of miR-133b loss or gain-of-function. Thus, *tppp3* can be defined as a new regulator of axon regeneration, at least in zebrafish M-cells.

To figure out whether miR-133b has an effects on mitochondrial motility or not, we performed an experiment to visualizing mitochondrial motility in miR-133b overexpression group, as mitochondrial dynamics has shown to have a positive correlation with regenerative capability (Zhou et al., 2016; Xu et al., 2017). Consistent with our axonal regeneration data, motile mitochondria rate and mitochondrial velocity were both decreased accompanying worsening regenerative capability upon miR-133b overexpressing. While the mechanism on this finding needs to be further explored, this result that miR-133b reduces mitochondrial dynamics, at least, further reinforces our conclusion that miR-133b diminishes regenerative capacity in M-cells.

The role of dre-miR-133b in regeneration appears context-dependent in different organs (Yin et al., 2008, 2012; Yu et al., 2011; Xin et al., 2013). Similar to the adverse function of miR-133b during M-cell regeneration process, it inhibits fin regeneration in adult zebrafish by targeting Mps1 (Yin et al., 2008) and negatively regulates zebrafish heart regeneration via restricting injury-induced cardiomyocyte proliferation (Yin et al., 2012). Also, miR-133b can enhance axon regeneration and promote functional recovery after SCI in zebrafish and mice by targeting RhoA (Yu et al., 2011; Theis et al., 2017). As for the divergence between our results and the results showing miR-133b can promote regeneration after SCI by targeting RhoA, one plausible explanation might be related to the different modes of injury. We regulated the expression of miR-133b at single-cell level and severed axons by two-photon laser axotomy, which only damaged axon at a minuscule area, separating the intracellular and intercellular factors influencing axon regeneration *in vivo* and reflecting the intrinsic role of miR-133b during axon regeneration process. For SCI, a complete transection of the spinal cord was carried out, which inevitably damaged a large number of neurons and extracellular milieu. Since miR-133b has been proved to reduce the activated microglias/microphoges at injury site (Theis et al., 2017), it is possible that miR-133b enables the neurons a higher regenerative capacity after SCI by, to some degree, playing a significant role in diminishing the inhibitory extracellular milieu. In addition, it has been proved that miR-133b enhance neurite outgrowth in cultured neurons (Lu et al., 2015; Theis et al., 2017). Cultured neurons are, however, developing cells, which normally stemmed from embryos or newborn animals, and axon growth occurs from the cell body rather than from the tip of a damaged axon. As axons only contain a subset of molecules that are found in the cell body, outgrowth from the soma may have different underlying biology to that of regeneration from the end of a cut axon (Bradke et al., 2012). Moreover, we cannot totally deny that miR-133b might play a role in differentiation in cultured neurons and miR-133b has been reported to promote differentiation process via ERK 1/2 pathway (Sanchez-Simon et al., 2010; Feng et al., 2013). Thus, as we focus on miR-133b's role during regeneration process of M-cells in our experiments, which has been mature during our experimental time window, we believe our conclusion of miR-133b inhibiting M-cell axon regeneration does not conflict with the conclusions mentioned above.

A large number of studies have demonstrated the critical role of miRNAs in regeneration process, however, many reports explore the function of miRNA in cell populations, masking the important information connecting single cell fate and miRNA function in it (Verdú et al., 2000). Studying miRNA role in one single cell is important because it allows deep understanding of the correlations between the miRNAs and cell function (Meacham and Morrison, 2013; Wills et al., 2013). In order to have a comprehensive understanding of miRNA function, we built a model to identify the miRNA function in zebrafish Mauthner cell regeneration by single-cell electroporation, presenting a new method to understand intrinsic miRNA function in regenerative process, without concerning with effects from intercellular context. Through combining effectively with other gene interference technology and subcellular organization mitochondria labeled by single-cell electroporation, we provided a new tool to explore functions of different genes in single cell *in vivo*.

In summary, our study identifies miR-133b as cell-intrinsic inhibitor of axon regeneration, which performs its function, at least partly, via regulating *tppp3* (Figure 7). These results, together with our single cell analysis approach, not only contribute significantly to the fundamental understanding of miRNA regulation in regeneration, but also have implications in developing therapeutic strategies for nerve injury.

## Author contributions

Designed the experiments: RH, MC, and BH. Performed the experiments: RH, MC, and LY. Contributed critical reagents: MW and SG. Analyzed the data: RH and MC. Wrote the manuscript: RH. Revised the manuscript: BH and SG.

### Conflict of interest statement

The authors declare that the research was conducted in the absence of any commercial or financial relationships that could be construed as a potential conflict of interest.

## Supplementary material

Figure S1 miR-133b duplex inhibits M-cell regeneration **(A)** Confocal imaging of M-cell at 1 dpa (top) and 2 dpa (bottom). White asterisk: ablation point. Scale bar: 50 μm. **(B)** Regeneration length at 1 and 2 dpa. One days post-axotomy: One-way ANOVA, *P* = 0.2195. Two days post-axotomy: One-way ANOVA, *P* = 0.1847.

Figure S2 The design of miR-shRNAs targeting *tppp3*. **(A)** The sequences of five shRNAs targeting *tppp3*. The guide strands (bottom) are highlighted in dark blue. **(B)** The location of shRNA target sites in the *tppp3* mRNA.

Video S1 *In vivo* imaging of mitochondrial movement in control Axonal mitochondrial motility along M-cell axon labeled with mito-EGFP and mCherry. 2.5-min time-lapse images were acquired with a 60 × lens and recorded for a total of 100 frames at 1.5-s intervals.

Video S2 *In vivo* imaging of mitochondrial movement in miR-133b overexpressed group Axonal mitochondrial motility along M-cell axon overexpressing miR-133b. M-cell was labeled with mito-EGFP and mCherry-miR-133b. 2.5-min time-lapse images were acquired with a 60 × lens and recorded for a total of 100 frames at 1.5-s intervals.
