# Supplementary figures and images for: MicroRNA-133b Negatively Regulates Zebrafish Single Mauthner-Cell Axon Regeneration through Targeting tppp3 in Vivo
Source: Front Mol Neurosci. 2017 Nov 21;10:375. doi: 10.3389/fnmol.2017.00375 (PMC5702462; doi:10.3389/fnmol.2017.00375)

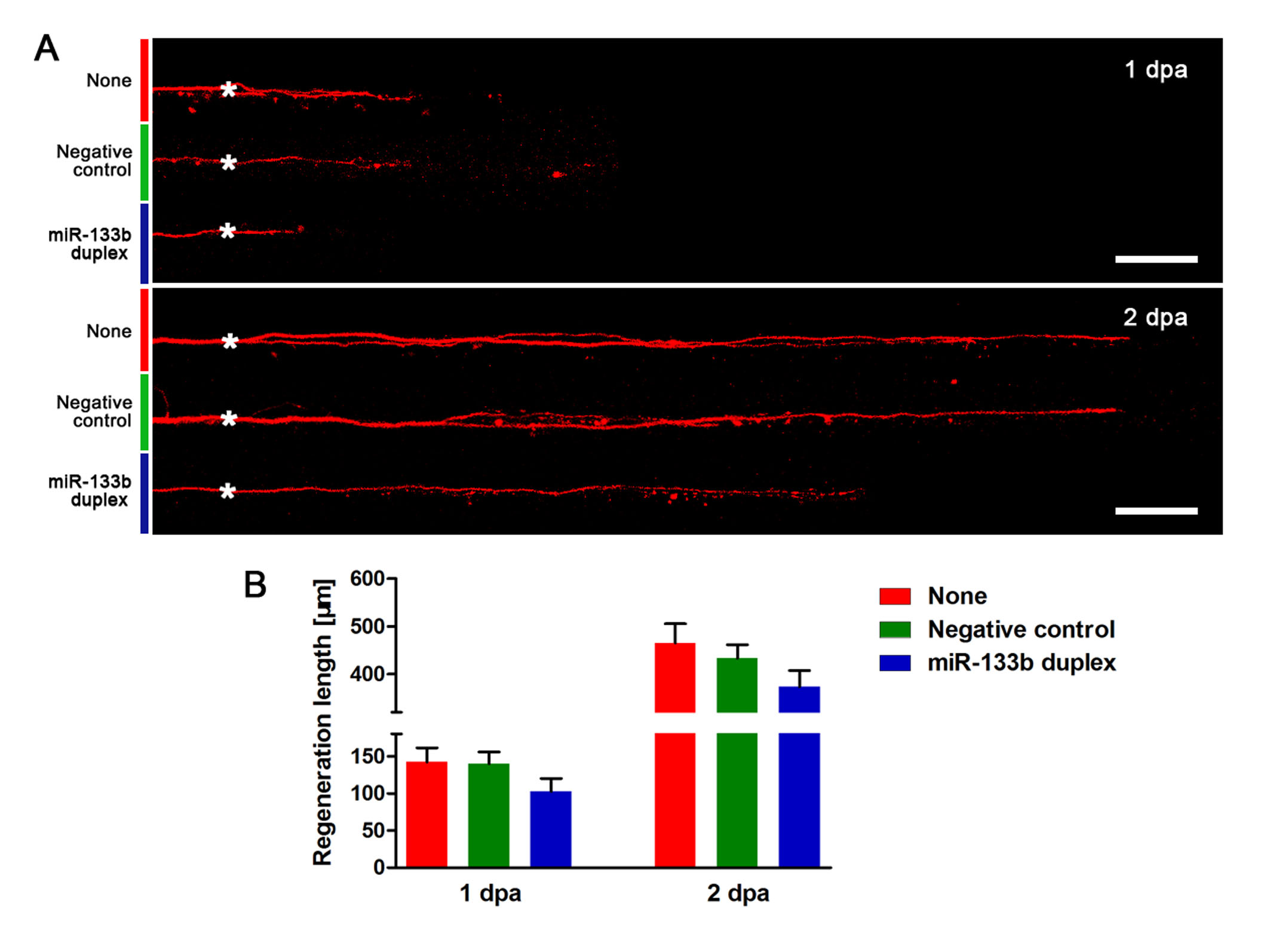

Supplement: Figure S1 — miR-133b duplex inhibits M-cell regeneration (A) Confocal imaging of M-cell at 1 dpa (top) and 2 dpa (bottom). White asterisk: ablation point. Scale bar: 50 μm. (B) Regeneration length at 1 and 2 dpa. One days post-axotomy: One-way ANOVA, P = 0.2195. Two days post-axotomy: One-way ANOVA, P = 0.1847. [file Image1.JPEG]

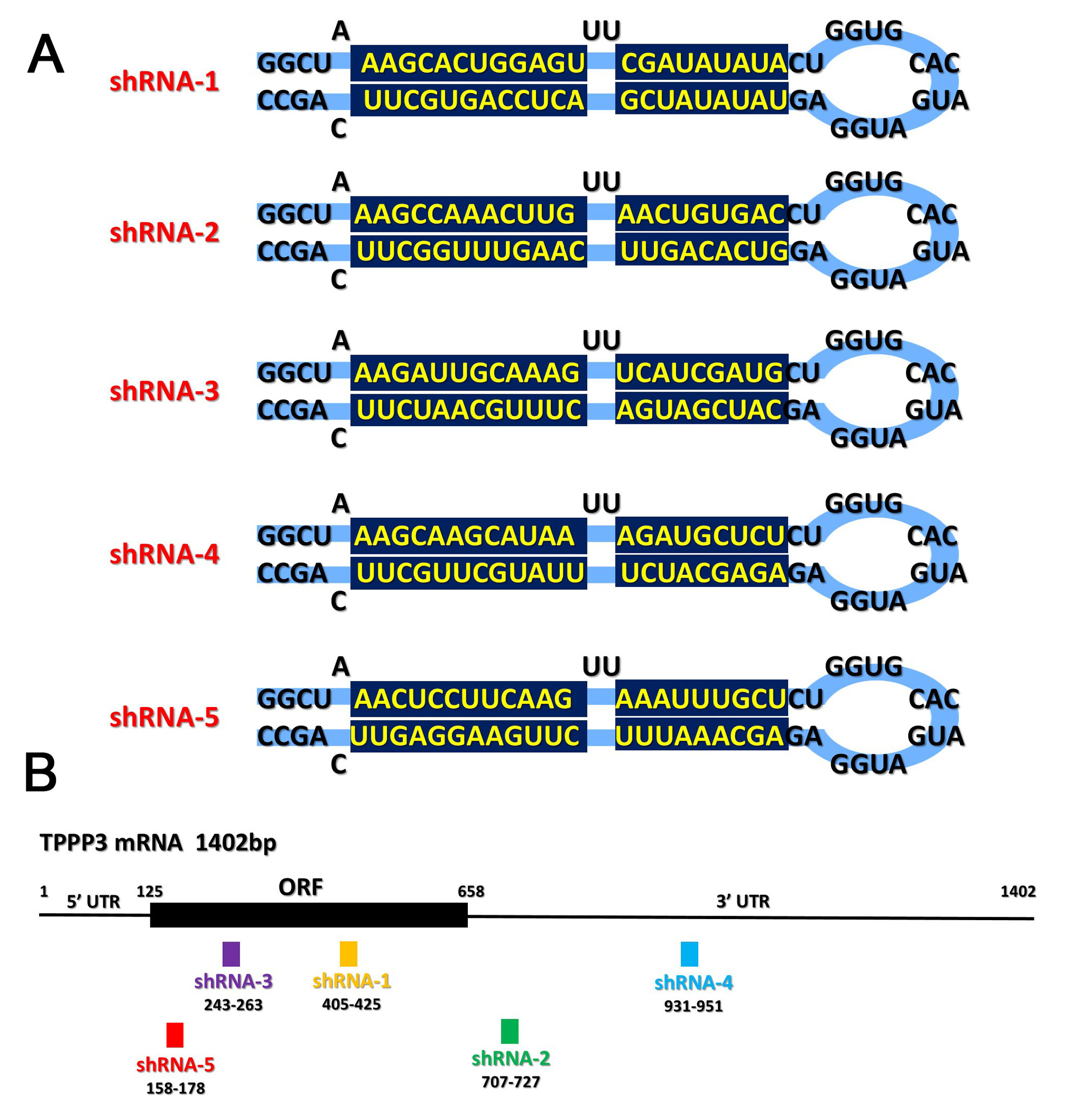

Supplement: Figure S2 — The design of miR-shRNAs targeting tppp3. (A) The sequences of five shRNAs targeting tppp3. The guide strands (bottom) are highlighted in dark blue. (B) The location of shRNA target sites in the tppp3 mRNA. [file Image2.JPEG]
