# Supplementary material for: MicroRNA-133b Negatively Regulates Zebrafish Single Mauthner-Cell Axon Regeneration through Targeting tppp3 in Vivo
Source: Front Mol Neurosci. 2017 Nov 21;10:375. doi: 10.3389/fnmol.2017.00375 (PMC5702462; doi:10.3389/fnmol.2017.00375)
Supplement: Supplementary file 5 [file Table1.DOCX]

**Table 1: qRT-PCR primers**

| Q-miR-133b-F | GCTGGTCAAATGGAACCAAGTC |
| --- | --- |
| Q-miR-21-F | TAGCTTATCAGACTGGTGTTGGC |
| Q-miR-23a-F | ATCACATTGCCAGGGATTTCCA |
| Q-U6-F | ATGACACGCAAATCCGTGAAG |
| Q-β-actin-F | CATTGGCAATGAGCGTTTC |
| Q-β-actin-R | TACTCCTGCTTGCTGATCCAC |
| Q-mps1-F | TGACGTGACCAGCATCATGAAGGA |
| Q-mps1-R | TGGAGGACGTGTCTTTAATGGCCT |
| Q-tppp3-F | AACACAGAATACCCAACATGGCAG |
| Q-tppp3-R | GTGCTGGTGACGTTCTTGCC |
